# Mining the Gene Wiki for functional genomic knowledge

**DOI:** 10.1186/1471-2164-12-603

**Published:** 2011-12-13

**Authors:** Benjamin M Good, Douglas G Howe, Simon M Lin, Warren A Kibbe, Andrew I Su

**Affiliations:** 1 Department of Molecular and Experimental Medicine, The Scripps Research Institute, 10550 North Torrey Pines Road, La Jolla, CA 92037, USA; 2 The Zebrafish Model Organism Database, University of Oregon, 5291, University of Oregon, Eugene, OR 97403, USA; 3 Department of Biomedical Informatics, Northwestern University, 750 North Lake Shore Drive, Chicago, IL 60611, USA

## Abstract

**Background:**

Ontology-based gene annotations are important tools for organizing and analyzing genome-scale biological data. Collecting these annotations is a valuable but costly endeavor. The Gene Wiki makes use of Wikipedia as a low-cost, mass-collaborative platform for assembling text-based gene annotations. The Gene Wiki is comprised of more than 10,000 review articles, each describing one human gene. The goal of this study is to define and assess a computational strategy for translating the text of Gene Wiki articles into ontology-based gene annotations. We specifically explore the generation of structured annotations using the Gene Ontology and the Human Disease Ontology.

**Results:**

Our system produced 2,983 candidate gene annotations using the Disease Ontology and 11,022 candidate annotations using the Gene Ontology from the text of the Gene Wiki. Based on manual evaluations and comparisons to reference annotation sets, we estimate a precision of 90-93% for the Disease Ontology annotations and 48-64% for the Gene Ontology annotations. We further demonstrate that this data set can systematically improve the results from gene set enrichment analyses.

**Conclusions:**

The Gene Wiki is a rapidly growing corpus of text focused on human gene function. Here, we demonstrate that the Gene Wiki can be a powerful resource for generating ontology-based gene annotations. These annotations can be used immediately to improve workflows for building curated gene annotation databases and knowledge-based statistical analyses.

## Background

In 2011, PubMed surpassed 21 million total articles in its index and is growing at a rate approaching 1 million new articles per year. Tools that make effective use of this knowledge base are increasingly vital to biomedical research [1]. Search interfaces like PubMed and Google Scholar help to find individual documents, yet no single document contains all of the knowledge about a particular biological concept. The knowledge is distributed throughout the text of many different articles with each new contribution simply adding to the stack. In considering the task of comprehensively capturing and utilizing society's biological knowledge, it is clear that document-centered approaches are insufficient.

Recognizing this problem and its clear pertinence to genome-scale biology, the research community began defining ontologies to capture and structure functional genomic knowledge even before the first human genome was fully sequenced [2-4]. Ontologies, such as the Gene Ontology (GO), provide a mechanism to efficiently bring together individual atomic facts (e.g. 'the product of the ABCA3 gene localizes to the plasma membrane') that may be scattered across many different texts. Such integration has enabled the production of new tools for interacting with and computing with massive bodies of knowledge. In particular, ontology-based computational analysis now plays a crucial role in the interpretation of the results of high-throughput, genomic studies [5,6].

While structuring knowledge using ontologies has proven highly beneficial, it presents some substantial challenges. The task of manually representing knowledge with an ontology is difficult, time-consuming and generally not rewarded by the scientific community. Current paradigms drive scientists to publish their findings as text in traditional journals that must subsequently be sifted through by separate teams of database curators who identify and extract new ontology-based facts. This process results in a significant bottleneck that is costly both in terms of the resources required and the likelihood that the system is not capturing all of the knowledge that is produced [7]. In addition, there remains a secondary challenge of presenting this knowledge to biologists of all levels in a manner that they can rapidly understand.

Faced with these challenges in manual biocuration, database curators have begun to investigate wikis as a potential solution [8,9]. Wikis represent a third approach to knowledge management that straddles the line between document-centric traditional publishing and ontology-driven knowledge base development. In contrast to typical literature collections, wikis, like ontologies, are concept-centric. When new facts are added, they are placed directly into the context of existing articles. For each gene, for example, a single wiki article can summarize knowledge spread over a large and growing corpus of traditional publications related to that gene. This concept-centricity renders wikis an excellent medium to capture rapidly evolving biological knowledge.

The other distinguishing attribute of wikis is their potential to make use of community intelligence on a massive scale. Wikipedia is well known for harnessing the intellects of literally millions of people to assemble the world's largest encyclopedia. As a result of both their concept-centric structure and their enticing potential to facilitate mass collaboration, wikis have emerged in a variety of areas of biology. We have wikis that capture information about genes [10-12], proteins [13], protein structures [14,15], SNPs [16], pathways [17], specific organisms [18] and many other biological entities.

In some cases, wikis are already successfully tapping into the community's collective intellect to produce useful biological knowledge repositories. One prominent example is the Gene Wiki [10,11]. The Gene Wiki is a growing collection of Wikipedia articles, each of which is focused specifically on a human gene. As of January 4, 2011, it contained articles on 10,271 human genes. These articles collectively amount to over 75 megabytes of text and more than 1.3 million words. In addition, they contain direct citations to more than 35,000 distinct articles in PubMed. To emphasize the collaborative scale of the Gene Wiki project, in 2010, these articles were edited by more than 3,500 distinct editors and were viewed more than 55 million times.

The Gene Wiki successfully harnesses community intelligence and escapes the "infinite pile" of document-centric approaches by maintaining a single, dynamic article for each gene. However, most of the captured knowledge is unstructured text and therefore it does not provide the structured gene annotations needed to effectively compute with the knowledge it contains. Wikipedia and the Gene Wiki are simply not designed to capture ontology-based facts. Hence, while the wiki-model can successfully summarize the collective knowledge of the community, the challenge of fully structuring the information remains.

Computational tools for finding ontology terms in text, such as the National Center for Biomedical Ontology's (NCBO) Annotator [19] and the National Library of Medicine's MetaMap [20], can help to address the challenge of structuring information presented in natural language. In this article we describe an approach for mining ontology-based annotations for human genes from the text of the Gene Wiki. Specifically we used the NCBO Annotator to identify structured gene annotations based on the Disease Ontology (DO) [21] and the Gene Ontology (GO) [4]. We evaluated the predicted annotations through comparison to known annotations and manual expert review. In addition, we assessed the impact of these predicted annotations on gene set enrichment analyses.

## Results

### System design

**Figure 1:**
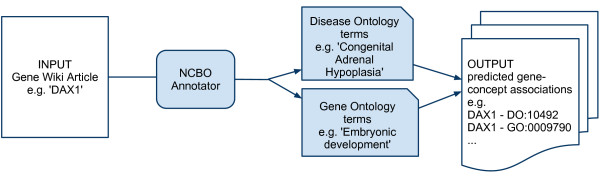
**Annotation mining from the Gene Wiki**. Text from Gene Wiki articles is processed with the NCBO Annotator to identify occurrences of terms from the Disease Ontology and the Gene Ontology. The result is a list of candidate associations between the gene described in the article and concepts from the ontologies.

As illustrated in Figure 1, we applied the NCBO Annotator to identify occurrences of DO and GO terms in Gene Wiki articles. The text of each Gene Wiki article was first filtered to eliminate references and auto-generated text, and then sent to the Annotator for concept detection. Since each article is specifically about a particular human gene, we made the assumption that occurrences of concepts within the text of a gene-centric article were also about the gene and considered such occurrences candidate annotations for the gene. For example, we identified the GO term 'embryonic development (GO:0009790)' in the text of the article on the DAX1 gene: "DAX1 controls the activity of certain genes in the cells that form these tissues during embryonic development". From this occurrence, our system proposed the structured annotation 'DAX1 participates in the biological process of embryonic development'. Following the same pattern, we found a potential annotation to the DO term 'Congenital Adrenal Hypoplasia' (DOID:10492) in the sentence: "Mutations in this gene result in both X-linked congenital adrenal hypoplasia and hypogonadotropic hypogonadism".

Overall, this workflow resulted in 2,983 candidate DO annotations (Additional File 1) and 11,022 candidate GO annotations (Additional File 2) from the collaboratively authored text of 10,271 Gene Wiki articles. We next characterized these candidate annotations through comprehensive comparison to pre-existing reference gene annotation databases and through manual inspection by experts in GO and DO annotation.

### Comparisons to Reference Annotations

Each candidate annotation was compared to reference annotations for the relevant gene. Matches to the reference could either be exact matches, matches to a more specific term in the same lineage as a reference annotation (with respect to the ontology's hierarchy) or matches to a more general term. For example, our system suggested that the Thrombin gene be annotated with 'hemostasis' (GO:0007599); since Thrombin was already annotated with 'blood coagulation' (GO:0007596) which is a narrower term than 'hemostasis' and in the same lineage, the predicted annotation was classified as a match to a more general term. (Additional details regarding the processing of the ontology hierarchies are found in the Methods section.)

#### Disease Ontology

We compared the candidate DO annotations to a pre-existing gene-disease annotation database mined from NCBI's GeneRIFs [21]. While Online Mendelian Inheritance in Man (OMIM) is probably the most widely recognized gene-disease database, we chose the GeneRIF-backed database for comparison because a) it used the DO for annotations thus enabling direct comparison (OMIM does not use any structured vocabulary for its disease annotations), b) at the time it was created, it contained the majority of the gene-disease associations in OMIM and significantly extended this set, and c) it reported a very high precision rate (96.6%) - comparable to a manually curated resource [21]. The downside of using this database for comparison was that it had not been updated since 2008 and hence it was undoubtedly missing more recent information.

**Figure 2:**
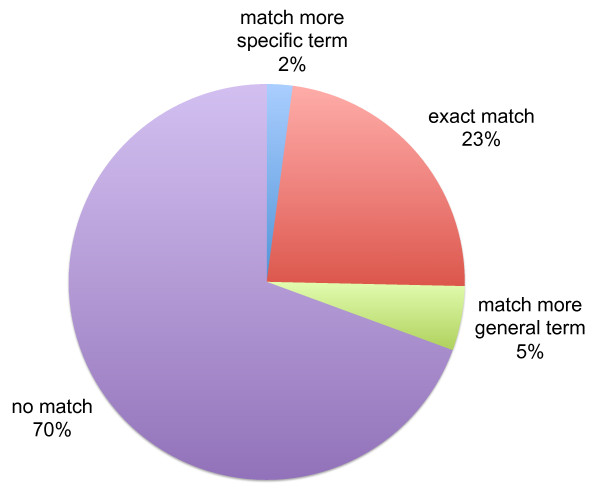
**Comparison of candidate Disease Ontology gene annotations mined from the Gene Wiki with annotations mined from GeneRIFs**. In total, 2,983 candidate Disease Ontology annotations were mined from the Gene Wiki. 23% of these matched annotations identified within GeneRIFs by (Osborne et al 2009), 2% matched a more specific term than a GeneRIF-based annotation, 5% matched a more general term, and 70% had no match.

In all, 693 (23%) of the 2,983 candidate annotations exactly matched an annotation from the DO reference annotations, 157 (5%) matched a more general term in the same lineage as a reference annotation, 63 (2%) matched a more specific term, and 2070 (70%) had no match (Figure 2). We refer to the annotations with no match as 'novel candidates' as these represent potential new annotations.

#### Gene Ontology

We next compared the candidate annotations to reference annotations from the GO annotation database (GOA) [22]. The GOA database is the accepted standard public reference for GO-based annotation of human gene products. When this analysis was conducted, it provided annotations for 17,940 distinct human genes. Of the 11,022 mined GO annotations, 1,853 (17%) matched an annotation in the GOA database exactly, 218 (2%) matched more specific terms than GOA annotations, 2,850 (26%) matched more general terms, and 6,101 (55%) did not match any.

The GO is divided into three distinct topical branches: Biological Process (BP), Molecular Function (MF) and Cellular Component (CC). In all, 54% of the 11,022 candidate annotations used BP terms, 14% used MF, and the remaining 32% used CC. In the remainder of this article, we focus on the 5,978 candidate BP annotations because they were the most plentiful in the output and because they are generally the most difficult annotations to determine automatically using other methods [23].

**Figure 3:**
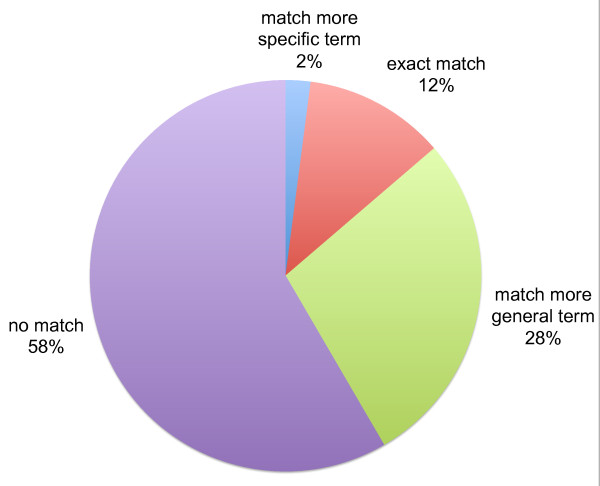
**Comparison of candidate Gene Ontology Biological Process annotations mined from the Gene Wiki to annotations from the GOA database**. In total, 5,978 candidate Gene Ontology annotations were mined from the Gene Wiki. 12% of these matched existing GOA annotations exactly, 2% matched a more specific term than a GOA annotation, 28% matched a more general term, and 58% had no match.

Of the 5,978 candidate BP annotations, 697 (12%) were direct matches to gene-function annotations in the GOA reference, 123 (2%) matched narrower terms than existing annotations, 1,667 (28%) matched more general terms than existing annotations, and the remaining 3,491 (58%) did not match any annotations in the GOA database (Figure 3).

### Manual Evaluations

For candidate annotations with no matches in the reference databases, we extracted a random sample and submitted it to expert curators to manually evaluate the quality of the predictions. For the DO evaluations, the sample contained 213 candidate annotations or approximately 10% of the 2,133 candidate annotations that either had no match in the reference set or matched a child of a reference annotation. For the GO we originally selected 200 novel candidate annotations for the evaluation but later had to remove 4 from consideration after discovering that, due to an error in processing, these were actually parents of reference annotations. The sample sizes were the largest that could be processed in a reasonable amount of time based on the curator resources at our disposal. For the evaluation, the curators assigned each candidate annotation in the sample to one of eight categories as follows.

Category 1: Yes, this would lead to a new annotation

**1A: perfect match **- the candidate annotation is exactly as it would be from a curator (e.g., Titin → Scleroderma)

**1B: not specific enough **- the candidate annotation is correct but a more specific term should be used instead (e.g., Titin → Autoimmune disease)

**1C: too specific **- the candidate annotation is close to correct, but is too specific given the evidence at hand (e.g., Titin → Pulmonary Systemic Sclerosis)

**Category 2: Maybe, but insufficient evidence**:

**2A: evaluator could not find enough supporting evidence in the literature after about 10 minutes of looking **(e.g., DUSP7 → cellular proliferation; there is literature indicating that DUSP7 is a phosphatase that dephosphorylates MAPK, and hence may play a role in regulating cell proliferation stimulated through MAPK. Although no direct evidence supporting this contention for Human DUSP7 was found, it seems plausible.)

2B: there is disagreement in the literature about the truth of this annotation

Category 3: No, this candidate annotation is incorrect:

**3A: incorrect concept recognition **(e.g., "Olfactory receptors share a 7-transmembrane domain structure with many neurotransmitter and hormone receptors and are responsible for the recognition and G protein-mediated transduction of odorant signals." [24] The system incorrectly identifies 'transduction' (GO:0009293) which is defined as the transfer of genetic information to a bacterium from a bacteriophage or between bacterial or yeast cells mediated by a phage vector - a completely different concept from *signal transduction *as intended in the sentence.)

**3B: incorrect sentence context **- the sentence is a negation or otherwise does not support the predicted annotation for the given gene (e.g., "The protein is composed of ~300 amino acid residues and has ~30 carbohydrate residues attached including 10 sialic acid residues, which are attached to the protein during posttranslational modification in the Golgi apparatus." [25] Such sentences may lead to incorrect candidate annotations of 'Golgi apparatus' and 'Posttranslational modification'.)

**3C: this sentence seems factually false **(e.g., a hypothetical example: "Insulin injections have been shown to cure Parkinson's disease and lead to the growth of additional toes".)

#### Disease Ontology

To assess the quality of the candidate DO annotations with no match in the reference set discussed above, we reviewed the 213 randomly selected novel candidate annotations manually according to the criteria outlined above (Additional File 3). The reviewers (authors SML and WAK) were experts in DO-based gene annotation and are active participants in the development of the DO.

**Figure 4:**
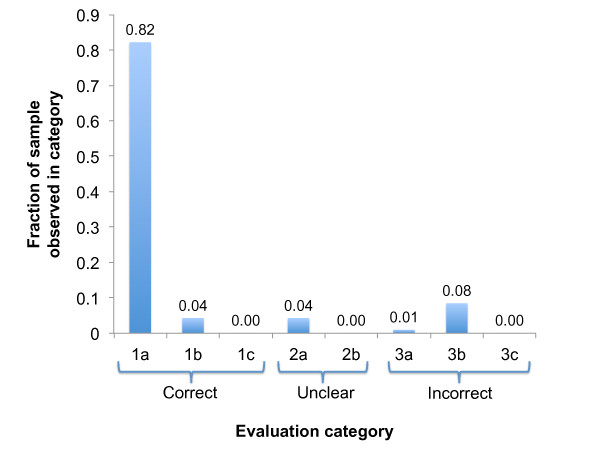
**Results of manual evaluation of novel candidate Disease Ontology annotations**. A random sample of 213 candidate annotations were evaluated. The evaluation categories were 1a: correct, 1b: correct but more general, 1c: correct but too specific, 2a: might be correct can't find evidence, 2b: might be correct, literature disagrees, 3a: incorrect concept recognition, 3b: incorrect sentence context, 3c: sentence context is factually false.

Out of the 213 candidates evaluated, 193 (91%) were classified in category 1 (yes, this would lead to a new annotation) with 175 (82%) assigned to category 1A (perfect match). Figure 4 provides a breakdown of the results of the manual evaluation. Nearly all of the errors fall into category 3B (incorrect sentence context). For example, the NCBO Annotator correctly identified the disease term 'neuroblastoma' in the following sentence: "Dock3-mediated Rac1 activation promotes reorganisation of the cytoskeleton in SH-SY5Y neuroblastoma cells and primary cortical neurones as well as morphological changes in fibroblasts"; however, the assumption that the occurrence indicates an association with the gene Dock3 does not hold because the sentence is referring to a neuroblastoma cell line rather than to the human disease.

#### Gene Ontology

**Figure 5:**
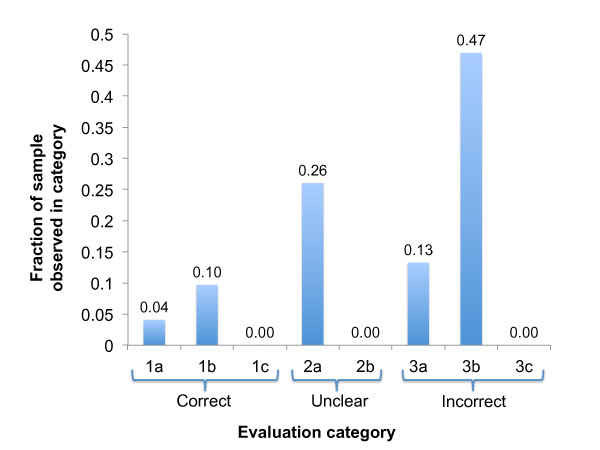
**Results of manual evaluation of novel candidate Gene Ontology annotations**. A random sample of 196 candidate Biological Process annoations were evaluated. The evaluation categories were 1a: correct, 1b: correct but more general, 1c: correct but too specific, 2a: might be correct can't find evidence, 2b: might be correct, literature disagrees, 3a: incorrect concept recognition, 3b: incorrect sentence context, 3c: sentence context is factually false.

We then followed the same protocol to assess the quality of candidate biological process annotations with no match in the GOA reference set (Additional File 4). A professional curator familiar with GO annotation (author DGH) manually inspected a random sample of 196 candidate annotations with no match in the reference set and classified each with the same 8 categories used for the DO evaluations. The performance was substantially worse for the novel GO annotations than for the DO (Figure 5) - in particular only 8 (4%) of the 196 candidates that were evaluated were assigned to category 1A (perfect match). Aside from the low number of 'exact match' results, candidate GO annotations generated many more uncertain results (26% 2A) as well as a very large fraction of errors due to incorrect sentence context (47% 3B).

It is worth noting that not one of errors detected in either the DO or the GO annotations were classified as 3C (sentence is factually false). This provides evidence that the text of the Gene Wiki (at least the approximately 400 sentences examined manually here) is consistently correct.

### Overall precision of annotation mining system

We integrated the results from both the manual assessments and the comparison to reference data sets to provide an estimate of the overall likelihood that a predicted annotation is biologically valid. We refer to this likelihood as 'precision' where precision (also known as positive predictive value) is equal to the ratio of true positive predictions to the sum of true and false positive predictions. In order to calculate precision, we need to divide all predictions into two classes 'true' and 'false'. For this analysis, the true positive set contained the direct matches to reference annotations, the matches to parents of reference annotations (though not as specific as they could be, these are still valid annotations), and the estimated number of Category 1 (would result in a new annotation) novel annotations. The estimated counts for the novel annotations were derived by multiplying the precision rates observed in the manually evaluated sample by the total number of novel candidate annotations. To account for the novel predicted annotations that were classified into Category 2A (maybe), we provide two estimates of precision: one that includes the 2A results as true positives and one that includes them as false positives. In this way we produce an estimated lower and upper bound on the system's actual precision. The estimated upper bound for the overall precision of the annotation protocol was thus calculated as:

(1) $$\frac{\left(exact+moregeneral+\left(\frac{e1a+e1b+e1c+e2a}{eAll}\right)*\left(none+child\right)\right)}{all}$$

and the estimated lower bound as:

(2) $$\frac{\left(exact+moregeneral+\left(\frac{e1a+e1b+e1c}{eAll}\right)*\left(none+child\right)\right)}{all}$$

where *exact*, *more general*, *child*, and *none *correspond to agreements between candidate annotations and a reference set; *e1a*, *e1b*, *e1c *and *e2a *refer to evaluation categories 1A, 1B, 1C and 2A, and *eAll *refers to the total number of novel candidates evaluated.

Using equations 1 and 2, we estimated a range for precision of 90-93% for the DO annotations and 48-64% for the GO annotations. In retrospect, it may be more appropriate to remove the category 1C ('too specific') annotations from the 'biologically valid' grouping here, but, since there were no occurrences of this category in either the DO or the GO evaluations, this change would not impact the results of the present analysis.

### Potential applications in enrichment analysis

Given these estimates of precision, we next checked to see if the annotations produced here could be used immediately in applications relevant to biological discovery. Specifically we assessed the use of the new annotations in the context of gene-set enrichment analyses.

Gene-set enrichment analyses provide a knowledge-based statistical assessment of the important concepts related to a set of genes [5,6]. Since tools for performing enrichment analysis are noise tolerant (small numbers of annotation errors do not overly disrupt the analysis), but cannot function without annotations, the use of automatically derived annotations as an extension to curated annotations can provide increased power and flexibility in terms of which concepts can be detected. For example, if a sufficient body of relevant text can be identified for each gene in a study set, enrichment analysis can now be conducted using any of the ontologies present in the NCBO BioPortal using the NCBO Annotator [26]. The crux of this kind of analysis is the identification of a sufficient quantity of relevant text. Since each Gene Wiki article is exclusively *about *one gene, as opposed to a typical article indexed in PubMed that may mention many genes and processes in very specific contexts, the articles form a particularly useful corpus. While the Gene Wiki alone does not yet have enough content to warrant the use of annotations derived solely from its text in knowledge-based analyses, we hypothesized that it could be used as an extension to other sources of gene-centric text to improve the results of text-driven enrichment analyses.

#### Evaluation of mined GO annotations in gene-set enrichment analysis

To assess the potential value of the gene-wiki derived GO annotations, we measured their impact on a controlled gene set enrichment experiment based on the pattern introduced by LePendu *et al*. [27]. As an example, consider the GO term for 'muscle contraction' (GO:0006936). This GO annotation was associated with 87 genes in the GOA database. After blinding ourselves to the origin of this 87 gene list, we performed text mining on the titles and abstracts of the publications associated with those 87 genes. We expected to find the term 'muscle contraction' to be enriched relative to the background occurrence in all publications. However, when using article titles and abstracts alone, we found no such statistical enrichment (p = 1.0). In contrast, after adding Gene Wiki text to the corpus of publication titles and abstracts, the term 'muscle contraction' was highly enriched (Fisher's exact test, p = 1.22 × 10^-9^, odds ratio 81.8).

To confirm that this result was not an artifact, we performed a simulation in which, for each of 1,000 iterations, we selected 87 genes randomly from the set of genes with any GO annotations from the GOA database and performed the identical analysis. This provided a way to estimate the probability that we would observe this improvement from the addition of the Gene Wiki text for random gene sets. The higher this probability, the lower our confidence that the additional annotations provided by the Gene Wiki provided a real signal of value. In only 5 of the 1,000 simulation runs did the Gene Wiki-enhanced annotation set produce a significant P value (p < 0.01) when the PubMed-only set did not. In fact, for the random gene sets the Gene Wiki derived annotations were slightly more likely to make the P values worse (19/1000) though in most cases there was no impact at all. This simulation demonstrated that, as should be expected from results presented above, the annotations mined from the Gene Wiki are both non-random and are clearly correlated with annotations shared in curated databases. In addition, it provided empirical evidence that Fisher's exact test is appropriate in this situation (see Methods for additional discussion of the selection of the test statistic).

**Figure 6:**
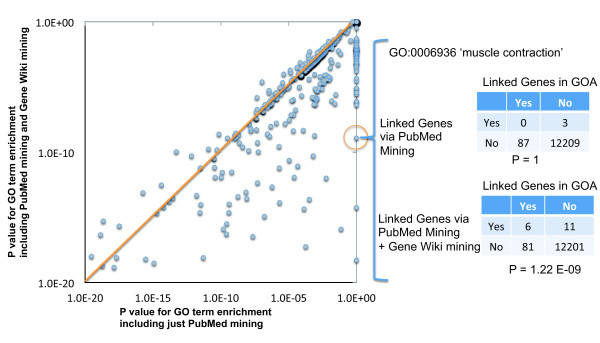
**Impact of Gene Wiki mined data on GO enrichment analysis**. Each point on the chart represents a test conducted on a single GO term. The X-axis presents the P values generated using just the annotations derived from PubMed text and the Y-axis depicts P values generated with the combination of the PubMed text and the Gene Wiki. (The few points with P values on either axis below 1.0E-20 are not shown to increase the visibility of the majority of the data.) Points below the orange line correspond to GO terms where the Gene Wiki text resulted in an improvement in the enrichment analysis. The data underlying the test on the GO term 'muscle contraction' are shown on the right.

We extended this analysis to all 773 GO terms used in human gene annotations and found a consistent improvement in the enrichment scores (Figure 6). Using abstracts and titles alone, this protocol resulted in a significant enrichment score for 314 gene sets (41%). Enhancing the data from PubMed mining with the Gene Wiki text resulted in an increase to 399 significant tests (52%).

The relatively low rate of annotation rediscovery for GO terms based on the text from abstracts related to associated genes is not surprising. Other groups have reported that only about 10% of the curated GO annotations can be found in the text of the abstract of the paper cited as evidence for the annotation [27]. What we show here simply demonstrates that the text from the Gene Wiki can extend the reach of systems that rely solely on the text of PubMed abstracts for annotation mining. While the results are preliminary, a similar text-driven enrichment analysis that used the DO rather than the GO also showed improvement when annotations mined from the Gene Wiki were included alongside annotations mined from PubMed abstracts (data not shown).

## Discussion

Our results demonstrate that the Gene Wiki is an important repository of knowledge about the human genome that is different from and complementary to other biological knowledge sources. Its articles provide a growing source of gene-specific text that pulls together relevant bits of information from the published literature into a form that is both useful for human consumption and highly amenable to natural language processing. Importantly, the mass collaborative approach to assembling these wiki articles scales with the explosive growth of the biomedical literature. Annotations mined from Gene Wiki text both recapitulated and extended knowledge in existing databases.

### Disease Ontology versus Gene Ontology for mining gene annotations

We speculate that the differences in the level of precision estimated for the predicted annotations using the GO (48-63%) and the DO (90-93%) were primarily the result of two key factors: the differing scopes of the two ontologies and differences in the way that the two communities view annotations. The extremely broad scope of the GO means that it has many fairly general terms, like 'transduction', that can result in errors due to polysemy. While fairly general terms do exist in the DO, such as 'dependence' (DOID:9973), they are far less frequent.

Aside from increased numbers of mismatched concepts from the GO, the criteria used by curators for establishing that a GO annotation is fit for inclusion in the GOA database are more stringent than the criteria for establishing a gene-disease association. This is evident when comparing the category 2A (maybe but insufficient evidence) results for the two ontologies. As shown in Figures 4 and 5, the reviewers felt that only 4% of the evaluated DO-based candidates needed additional investigation while 26% of the GO-based candidates were judged to require more evidence. In many cases, the 2A scores for the GO evaluations resulted when the main evidence for the new annotation was from research conducted in a model organism. In order for a GO curator to accept evidence from another organism, further analysis of sequence and phylogenies must be conducted and such analysis was beyond the scope of this evaluation. In the case of the DO annotations, curators accept evidence from model organisms as sufficient for forming an association.

### Potential modifications

It is worth noting that the basic protocol described here is not tied to the Annotator, it could be used with any concept detection system. The Annotator was used in this analysis because it has a fast convenient API, access to a large number of ontologies including the DO, and has been shown to have similar performance to MetaMap - a longstanding, commonly used tool to for biomedical concept recognition [28]. Improvements to the Annotator workflow, such as negation detection, are ongoing and will benefit the protocol described here. For critical assessments of other text-mining tools and applications in biology, see the BioCreative competitions [29]. In particular see [30] for a discussion of challenges in working with the GO.

In our evaluation of the results, we only had access to a single qualified GO curator and two DO curators, hence we had to constrain the size of the sample processed and could not calculate inter-annotator agreement (the DO curators discussed and resolved all discrepancies). While we suggest that the number of manually reviewed candidate annotations was sufficient to provide rough estimates on the precision of this protocol, additional evaluations would certainly be valuable. The scarcity of qualified curators, and the even more apparent scarcity of their time, provides additional motivation for continuing this area of research.

### Applications

We expect that predicted annotations from the Gene Wiki will have several applications.

First, professional biocurators could use Gene Wiki-derived annotations as a useful starting point for their curation efforts. Most obviously, these candidate annotations could be processed according to current curatorial standards (similar to the expert evaluation described in this study) to approve, refine, or reject them as formal annotations [31]. On an even more basic level, curators could simply prioritize PubMed articles that were used in inline Gene Wiki citations for formal review. In this scenario, the Gene Wiki would be used as a crowdsourced method to identify the most relevant scientific literature [32], an increasingly difficult problem based on the rapid growth of PubMed.

Second, these candidate annotations could be used directly by end users in statistical analyses that are tolerant to noisy data. For example, gene set enrichment analysis is among the most popular analysis strategies for genomic studies, and the underlying statistical test is, by definition, noise tolerant. A recent application called the Rich Annotation Summarizer (RANSUM) performs gene set enrichment analysis using any of the ontologies in the NCBO BioPortal by applying the Annotator to extract relevant annotations from MEDLINE abstracts and the NCBO Resource index [26]. Annotations derived from the Gene Wiki could fit directly into these and related systems.

## Conclusions

Ontology-based gene annotation forms a crucial component of many tasks in bioinformatics, but accumulating these annotations is costly. By combining the mass collaboratively generated text of gene-specific articles in the Gene Wiki with readily accessible natural language processing technology, we introduced a new and scalable system for generating gene annotations. As with any application of currently available natural language processing, this system is not error free. GO annotations in particular proved difficult to produce at high precision. Looking forward, we can expect that improvements in information extraction technology and the continued expansion of the gene-centric text in the Gene Wiki will combine to produce an increasingly valuable process for harnessing the ever-expanding body of functional knowledge about the human genome.

## Methods

### Gene Wiki Mining workflow

The wikitext from Gene Wiki articles was gathered using a Java program that accessed the data via the Wikipedia API [33]. For each article, the program processed the wikitext to:

1. identify references

2. identify individual sentences

3. identify the most recent author of each sub-block of each sentence where sub-blocks are determined by the sentence's edit history

4. remove most wikimarkup from the text

Following the wikitext processing, the extracted sentences were sent to the NCBO Annotator service for concept detection. Only sentences from the main article body (not the reference section) were sent to the Annotator. Table 1 describes the parameters used for the Annotator (see Annotator Web service documentation for details [34]). Note that these parameters were selected to maximize the precision of the results - only the longest, whole-word match for a text fragment was returned and matching was only performed against the preferred term for each ontology concept (no synonyms were considered). The reason that we chose to disallow synonyms was that, at the time the analysis was performed, the Annotator was using all of the synonyms provided by the source ontologies, but some of these synonyms were not truly synonymous. The GO uses four different kinds of synonyms, 'exact', 'broader', 'narrower', and 'related' [35]. In initial testing, non-exact synonyms were producing many incorrect concept matches and, since there was no way to indicate to the Annotator which kinds of synonyms to consider, we chose to not allow any. (This problem led to conversations with the NCBO team with the end result that the Annotator service now only processes 'exact' synonyms from the GO as these are the only true synonyms provided.) Including proper synonyms should expand the annotation recall of the approach described here without reducing precision.

**Table 1:** NCBO Annotator Parameters

Parameter	Value
filterNumber	true

minTermSize	3

withSynonyms	false

longestOnly	true

wholeWordOnly	true

stopWords	protein, gene

withDefaultStopwords	true

score	true

mappingTypes	null

ontologies used	GO, DO

### Filtering the mined annotations

The results from the Annotator were translated into candidate annotations for each Gene Wiki gene. In addition to the stopwords sent to the Annotator, we filtered out uninformative predicted annotations that used the GO terms 'chromosome, 'cellular component' and the DO terms 'disease', 'disorder', 'syndrome', and 'recruitment'. Using markup provided by the WikiTrust system [36], we linked each candidate annotation to the last Wikipedia author to edit the text from which it was extracted. We used this authorship information to remove annotations extracted from text that had been imported automatically from NCBI Gene summaries during the initial creation of the Gene Wiki articles. While this text contains useful information, we chose to remove it from this analysis because we wanted to focus on the text edited by Gene Wiki users.

An executable program for identifying GO and DO annotations in Wikipedia articles according to the protocol described above as well as all relevant source code may be accessed at the open source Gene Wiki code repository [37].

### Comparison of mined annotations to reference annotations

Figures 2 and 3 were generated by comparing each candidate gene-concept pair to annotations from a reference set. This comparison was enacted as follows; for each gene-concept pair predicted from the Gene Wiki:

1. find all the terms annotated to the gene in the reference annotation set (e.g. from the GOA database).

◦ (For the GOA comparisons, we included electronically generated annotations which use the IEA evidence code.)

2. find all the broader and narrower terms related to each of the reference annotations

◦ For the DO, use only 'is a' relationships for this expansion

◦ For the GO, use 'is a', 'part of', 'regulates', 'positively regulates', and 'negatively regulates'. Each of these relations is treated in the same way as an 'is a' relation. This ensures that we can identify when our system identifies terms that are closely related to the terms used in a reference annotation even when they are not linked through a subsumption relationship. (The versions of the DO used in this work do not make extensive use of non-'is a' relationships). For example, if we discover the term 'positive regulation of transcription from RNA polymerase II promoter' and there is a reference annotation to the term 'transcription from RNA polymerase II promoter' we would record this as a match to a narrower term than existing annotation though the relation between these terms is 'positively regulates'.

3. record when a predicted annotation for a gene matches a reference annotation exactly, when it matches a broader term than an existing annotation, when it matches a narrower term and when it does not have a match.

### Gene-set enrichment experiment

**Figure 7:**
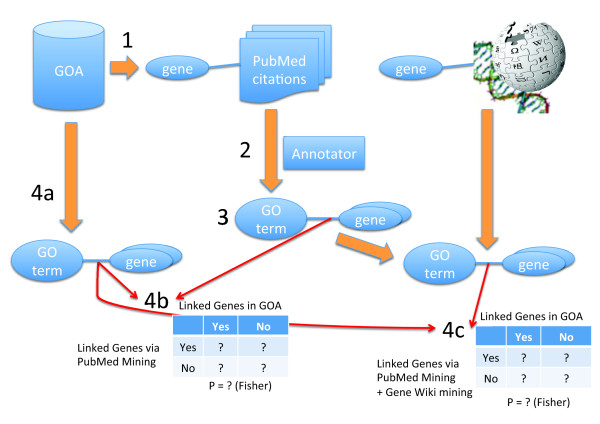
**Workflow for conducting controlled gene set enrichment experiment using the GOA database**. 1) PubMed citations are identified that are linked to genes in the context of GOA annotations. 2) The title and abstracts of each of these articles are mined for GO terms using the Annotator. 3) The mined terms are assigned as candidate annotations to the genes linked to the relevant PubMed citations. 4) For each GO term a) create a "true positive" gene set based on the annotations for that term from GOA, b) compare this gene set to the gene set produced for that GO term by the PubMed mining process, c) expand the PubMed-mined annotation set with the annotations mined from the Gene Wiki and repeat the comparison to the GOA-derived gene set. Compare the results from steps 4b and 4c to quantify the impact that the Gene Wiki-derived annotations have on gene set enrichment analysis where the 'correct' set is predetermined.

The protocol for the gene set enrichment experiment is depicted in Figure 7 and worked as follows.

1. Connections between genes and relevant PubMed citations are identified using literature-based records from the GOA database. The fact that a citation is used as evidence of gene function by a human curator provides good evidence that the text in the article is somehow about the gene [27].

2. The title and abstracts of each of these articles are mined for GO terms using the Annotator following the same protocol as applied to the Gene Wiki text.

3. The mined terms are assigned as candidate annotations to the genes linked to the relevant PubMed citations.

4. For each GO term used to create a non-IEA annotation for a human gene in the GOA database:

a. create a "true positive" gene set based on the non-IEA annotations for that term from GOA

b. compare this gene set to the gene set produced for that GO term by the PubMed mining process

• build a contingency table where the values in the cells are the numbers of genes assigned or not assigned to the GO term by GOA and by the PubMed mining system.

• assess the probability that the two gene sets are independent using Fisher's exact test

c. expand the PubMed-mined annotation set with the annotations mined from the Gene Wiki and repeat the comparison to the GOA-derived gene set

The results from steps 4b and 4c were compared to quantify the impact that the Gene Wiki-derived annotations had on gene set enrichment analysis (Additional File 5).

Note that Fisher's exact test is used in many widely used tools for conducting enrichment analysis including: DAVID, FatiGO, and GoMiner. As the situation modeled in this experiment is precisely that of an enrichment analysis, this provides evidence that this is a reasonable test statistic. Many of the tools that do not use Fisher's test report the use of the hypergeometric test, e.g. BINGO, CLENCH, FunSpec. This test is exactly equivalent to the one-tailed version of Fisher's test [38].

### Resources used

In this work we made extensive use of external APIs and datasets. Table 2 provides a list of the resources used, their URLs, and the date we accessed them.

**Table 2:** Resources used

Resource	Gathered from	Access Date
Gene Wiki Articles	http://en.wikipedia.org/w/api.php	Jan. 4, 2011

NCBO Annotator	http://www.bioontology.org/wiki/index.php/Annotator_Web_service	Jan. 12, 2011

WikiTrust	http://www.wikitrust.net	Jan. 11, 2011

OWL version of the Disease Ontology (for evaluation)	http://www.obofoundry.org/cgi-bin/detail.cgi?id=disease_ontology	Jan. 6, 2011

Disease Ontology annotations (from GeneRIF mining (Osborne et al 2009))	http://projects.bioinformatics.northwestern.edu/do_rif	Created Oct. 23, 2008. Accessed Jan. 7, 2011

OWL version of the Gene Ontology (for evaluation)	http://www.geneontology.org/GO.downloads.ontology.shtml (cvs version: $Revision: 1.1439)	Sept. 22, 2010

Gene Ontology Annotations	ftp://ftp.ncbi.nih.gov/gene/DATA/gene2go.gz (no version information provided) * we used all annotations including IEA in our analysis	Dec. 13, 2010

This table provides the name, URL, access date, and version number (when available) of the Web-based tools and databases used to conduct this study.

## List of Abbreviations

GO: Gene Ontology; DO: Human Disease Ontology; GOA: Gene Ontology Annotation database.

## Competing interests

The authors declare that they have no competing interests.

## Authors' contributions

BMG and AIS conceived of the study. BMG implemented all required code for producing and evaluating the candidate gene annotations and drafted the first version of the manuscript. DGH performed the manual evaluations of the candidate Gene Ontology annotations and contributed conceptually from the early phases of the study. SML and WAK conducted the manual evaluations of the candidate Disease Ontology annotations and contributed to the design of the evaluation process. All authors read, contributed to and approved the final manuscript.

## Supplementary Material

Additional file 1 **Additional data file 1 is a spreadsheet providing the candidate Disease Ontology annotations**. Click here for file

Additional file 2 **Additional data file 2 is a spreadsheet providing the candidate Gene Ontology annotations**. Click here for file

Additional file 3 **Additional data file 3 provides the results of the manual evaluations on the Disease Ontology sample**. Click here for file

Additional file 4 **Additional data file 4 provides the results of the manual evaluations on the Gene Ontology sample**. Click here for file

Additional file 5 **Additional data file 5 provides the data generated for the gene set enrichment experiment**. Click here for file
